# Risk factors, clinical correlates, and social functions of Chinese schizophrenia patients with drug-induced parkinsonism: A cross-sectional analysis of a multicenter, observational, real-world, prospective cohort study

**DOI:** 10.3389/fphar.2023.1077607

**Published:** 2023-03-03

**Authors:** Jiajun Weng, Lei Zhang, Wenjuan Yu, Nan Zhao, Binggen Zhu, Chengyu Ye, Zhanxing Zhang, Changlin Ma, Yan Li, Yiming Yu, Huafang Li

**Affiliations:** ^1^ Shanghai Mental Health Center, Shanghai Jiao Tong University School of Medicine, Shanghai, China; ^2^ Shanghai Zhongshan Hospital, Shanghai, China; ^3^ Shanghai Pudong District Mental Health Center, Shanghai, China; ^4^ Shanghai Minhang District Mental Health Center, Shanghai, China; ^5^ Shanghai Baoshan District Mental Health Center, Shanghai, China; ^6^ Shanghai Jiading District Mental Health Center, Shanghai, China; ^7^ Shanghai Clinical Research Center for Mental Health, Shanghai, China

**Keywords:** drug-induced parkinsonism, antipsychotics, schizophrenia, side effects, mood stabilizer

## Abstract

**Background:** Drug-induced parkinsonism (DIP) is the most prevalent neurological side effect of antipsychotics in the Chinese population. Early prevention, recognition, and treatment of DIP are important for the improvement of treatment outcomes and medication adherence of schizophrenia patients. However, the risk factors of DIP and the impact on the clinical syndromes of schizophrenia remain unknown.

**Aim:** The goal of this study was to explore the risk factors, clinical correlates, and social functions of DIP in Chinese schizophrenia patients.

**Methods:** A cross-sectional analysis of a multicenter, observational, real-world, prospective cohort study of the Chinese schizophrenia population with a baseline assessment was conducted from the year 2012 to 2018. Participants were recruited from four mental health centers in Shanghai and totaled 969 subjects. Sociodemographic data, drug treatment, and clinical variables were compared between the DIP group and the non-DIP group. Variables that correlated with the induction of DIP, and with *p*≤ 0.1, were included in the binary logistic model for analyzing the risk factors of DIP. First generation antipsychotics (FGA)/second generation antipsychotics (SGA) model and high and low/medium D2 receptor antipsychotics were analyzed respectively to control the bias of co-linearity. All risk factors derived from the a forementioned models and clinical variables with *p*≤ 0.1 were included in the multivariate analysis of clinical correlates and social function of DIP patients. The Positive and Negative Syndrome Scale (PANSS) model and the personal and social performance (PSP) model were analyzed separately to control for co-linearity bias.

**Results:** Age (OR = 1.03, *p*< 0.001), high D2 receptor antagonist antipsychotic dose (OR = 1.08, *p* = 0.032), and valproate dose (OR = 1.01, *p* = 0.001) were the risk factors of DIP. FGA doses were not a significant contributor to the induction of DIP. Psychiatric symptoms, including more severe negative symptoms (OR = 1.09, *p*< 0.001), lower cognition status (OR = 1.08, *p* = 0.033), and lower excited symptoms (OR = 0.91, *p* = 0.002), were significantly correlated with DIP induction. Social dysfunction, including reduction in socially useful activities (OR = 1.27, *p* = 0.004), lower self-care capabilities (OR = 1.53, *p*< 0.001), and milder disturbing and aggressive behavior (OR = 0.65, *p*< 0.001), were significantly correlated with induction of DIP. Valproate dose was significantly correlated with social dysfunction (OR = 1.01, *p* = 0.001) and psychiatric symptoms (OR = 1.01, *p* = 0.004) of DIP patients. Age may be a profound factor that affects not only the induction of DIP but also the severity of psychiatric symptoms (OR = 1.02, *p*< 0.001) and social functions (OR = 1.02, *p*< 0.001) of schizophrenia patients with DIP.

**Conclusion:** Age, high D2 receptor antagonist antipsychotic dose, and valproate dose are risk factors for DIP, and DIP is significantly correlated with psychiatric symptoms and social performance of Chinese schizophrenia patients. The rational application or discontinuation of valproate is necessary. Old age is related to psychotic symptoms and social adaption in Chinese schizophrenic patients, and early intervention and treatment of DIP can improve the prognosis and social performance of schizophrenia patients.

**Clinical Trial Registration:** Identifier: NCT02640911

## 1 Introduction

Extrapyramidal symptoms (EPS) are neurological side effects caused by antipsychotics that block the postsynaptic D2 receptors in the substantia-nigra/striatum pathway (Sachdev, 2005). The occurrence of EPS can make patients feel extremely uncomfortable and decrease medication compliance (Blair and Dauner, 1992; Leucht et al., 2013). The incidence of EPS remains substantial despite the use of second-generation antipsychotics (SGA) (Peluso et al., 2012; Divac et al., 2014).

Drug-induced parkinsonism (DIP) is deemed to be the most prevalent side effect among antipsychotic-induced movement disorders, ranging from 17% to 65.9% (Muscettola et al., 1999; Modestin et al., 2008; Musco et al., 2019), but the real incidence rate of DIP may be higher because of misdiagnosis or non-recognition of the symptoms (Friedman, 2014). It has been reported that antidepressants and mood stabilizers, including valproate, can induce EPS (Madhusoodanan et al., 2010; Revet et al., 2020; Zadikoffpoulos et al., 2021). However, whether antidepressants and mood stabilizers have an impact on the induction of DIP in the Chinese schizophrenia population remains unknown. The mismanagement of DIP can lead to the prolonged existence of symptoms and deterioration of motor function and physical condition of schizophrenia patients (Zadori et al., 2015). Some DIP patients may develop tardive dyskinesia (TD) which is a deteriorating hyperkinetic movement disorder affecting both the neuromotor and neurocognition functions of patients (Solmi et al., 2018). Proper management and prevention of DIP not only improves the clinical outcomes and physical condition of patients but also prevents the induction of TD in the early stages.

To the best of our knowledge, there have been few clinical trials with large samples reporting the prevalence, risk factors, clinical correlates, and social functions of DIP patients in the Chinese schizophrenia population. Only one article from our team focused on the prevalence, risk factors, and clinical correlates of overall EPS (Weng et al., 2019). We found that the use of a high-dose D2 antagonist antipsychotic was a high risk factor for EPS, but we did not explore the relationship between the D2 antagonistic effect and dose of the antipsychotic and the specific type of EPS. Given the enlargement of our study cohort and the problems that remain unsolved in previous studies, we decided to further analyze our database. The two main objectives of our study were to explore the prevalence and risk factors of DIP and to pursue the related clinical correlates and social functions of DIP in the real-world Chinese schizophrenia population.

## 2 Study participants and methods

### 2.1 Study design and participants

The multi-center, real-world clinical study of long-term outcomes for schizophrenia by atypical antipsychotic treatment (SALT-C) was an observational prospective cohort study that aimed to evaluate the safety of eight typical antipsychotic drugs frequently used in the treatment of schizophrenia in the Chinese population and to devise an optimum treatment plan for schizophrenia patients. The study design, inclusion criteria, exclusion criteria, clinical evaluation, pharmacological treatment plan, and additional information on SALT-C were published with the protocol of our study (Yu et al., 2021). To minimize bias, subjects with a history of neurological disease or brain trauma, or those addicted to alcohol or drugs such as benzodiazepine, were excluded in the data analysis of this article. The study protocol was approved by the ethical board of the Shanghai Mental Health Center (No 2016-23), and all procedures were performed in accordance with the ethical standards laid down in the 1964 declaration of Helsinki and later amendments. All subjects included in our study have signed a written informed consent. The clinical trial has been registered on the Clinical Trial website (NCT02640911).

**FIGURE 1:**
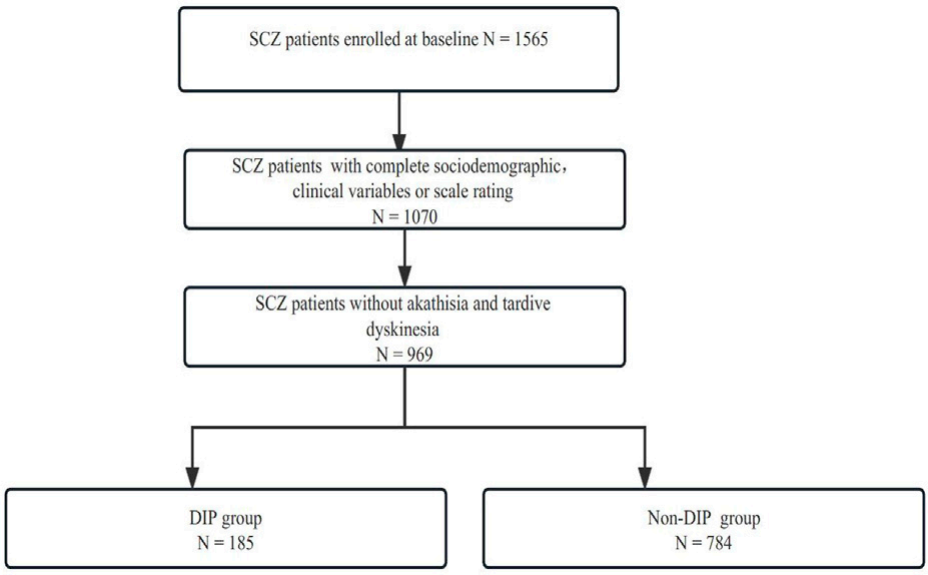
Detailed screening process of SALT-C in the present study.

The detailed flowchart of our trial is presented in Figure 1.We recruited 1542 subjects into the study. Because the main objective of the study was to explore the prevalence, risk factors, clinical correlates, and social functions of patients with DIP, those subjects without complete sociodemographic data, clinical variables, or scale ratings were excluded, along with those who had akathisia and TD. The diagnostic criteria of akathisia and TD are listed in the following paragraphs. Eventually, 969 subjects underwent the analysis.

### 2.2 Data collection

#### 2.2.1 Sociodemographic data and clinical variables

The sociodemographic data, including age and sex, were collected. The psychiatric symptoms were assessed according to the five-factor model of the Positive and Negative Syndrome Scale (PANSS) (Jiang et al., 2013), which is categorized into five sections: positive symptoms, negative symptoms, excitement, depression, and cognition. The depressive symptoms were evaluated with the Calgary Depression Rating Scale for Schizophrenia (CDSS) (Addington et al., 1990). This scale contains nine items and enables depression to be assessed independently of negative or EPS-related depressive phenomena in schizophrenia. The Clinical Global Impression-Severity (CGI-S) scale is a widely accepted tool that measures overall disease severity and the change of schizophrenia (Guy, 1976). The CGI comprises two linked one-item scales evaluating the severity of psychopathology on a scale of 1 to 7, and changes from the initiation of treatment on a similar seven-point scale. It is readily understandable and can be used with relative ease by the clinician.

The Personal and Social Performance (PSP) scale is used to evaluate the social functioning of schizophrenia patients (Morosini et al., 2000). The PSP scale contains four domains: (1) socially useful activities, (2) personal and social relationships, (3) self-care, and (4) disturbing and aggressive behaviors. Each domain is rated on a 6-point scale according to specific operational criteria to measure the severity of difficulties (1 = absent, 2 = mild, 3 = manifested but not marked, 4 = marked, 5 = severe, and 6 = very severe).

Current treatment, including types and doses of antipsychotics, mood stabilizers, and the use of additional drugs (antidepressants and anticholinergics), were recorded accordingly. The dosage of antipsychotics was transformed into a chlorpromazine equivalent dose (CPZ 100 mg) (Inada and Inagaki, 2015). To investigate the different receptor pharmacological mechanisms of antipsychotics that contribute to the induction of EPS, antipsychotics are classified as 1) FGA and SGA criteria, 2) antipsychotics with strong D2 receptor antagonism and slow D2 receptor dissociation ability are classified with high and the remaining antipsychotics are classified with low/medium D2 receptor antagonistic antipsychotic. The classification as SGA and FGA was based on the recommendations of a recent study and textbook (Divac et al., 2014; Wang et al., 2014). SGAs such as risperidone, ziprasidone, and paliperidone, and all FGAs, are classified as high D2 receptor antagonistic antipsychotics based on the recent conclusions of receptor pharmacology research and meta-analysis (Leucht et al., 2013; Sykes et al., 2017). The categories of the high and low/medium D2 receptor antagonistic effect antipsychotics are shown in Supplementary Material S1. Considering that long-term antipsychotics use may exert oxidative damage and dopaminergic neurotoxicity, which are important risk factors for EPS and Parkinson’s disease, the dosage of the high and low/medium D2 receptor antagonistic effect antipsychotics × years since the first prescribed antipsychotic and SGA/FGA × years since the first prescribed antipsychotic was included in our analysis (Miller et al., 2005; Zareifopoulos et al., 2021; Vaiman et al., 2022).

#### 2.2.2 Diagnostic criteria for EPS

The Simpson-Angus Scale (SAS) is a 10-item scale that is frequently used for assessing DIP in clinical practice. The score of each patient ranges from 0 to 40. According to the diagnostic criteria of CATIE, having six SAS items with a total score ≥2 are considered diagnostic for DIP (Simpson and Angus, 1970; Miller et al., 2005). Symptoms such as bradykinesia (item 1), arms dropping (item 2), shoulder shaking (item 3), elbow rigidity (item 4), wrist rigidity (item 5), and tremor (item 9), are included in the six-item SAS model.

The Barnes Rating Scale (BARS) is used for diagnosing akathisia (Barnes, 1989), and a BARS global item ≥2 is considered akathisia. The Abnormal Involuntary Movement Scale (AIMS) is used for diagnosing TD and is based on the Schooler and Kane criteria (a score of 2 on two items or a score of ≥3 on one item on the AIMS scale) (Schooler and Kane, 1982). Among our sample group, 65 patients with akathisia and 36 with TD were excluded from the baseline data of the study according to the diagnostic criteria.

#### 2.2.3 Statistical analysis

The sociodemographic and clinical variables, medications, clinical symptoms, and social functions were compared between the DIP group and the non-DIP group. A Kolmogorov–Smirnov test (K-S test) was conducted to test for normal distribution of the data. If the data distribution was non-normal, the groups were compared using the Mann-Whitney *U* test. If the data distribution was normal, the continuous variables of the groups were compared using Student’s *t* test. The chi-squared test was used for the comparison of categorical variables. A *p* ≤ 0.05 was considered statistically significant in a two-sided test. Logistic analysis was used for exploring the odds ratio (OR) and risk factors of DIP, clinical correlates, and social functions after adjusting for confounding factors (psychiatric symptoms and social functions were not included in the risk factor model because they cannot induce DIP). To discover more underlying risk factors of DIP, the variables were selected based on the broad-line significance of *p* ≤ 0.1 or clinical interest. Since anticholinergics were administered for symptomatic treatment after the induction of EPS, they were not included in the multivariate model. A correlation analysis was conducted to test the co-linearity bias between FGA dosage and high D2 receptor antagonistic antipsychotic dosage. If co-linearity bias existed, the dosage and usage of high and low/medium D2 receptor antagonistic antipsychotic dosage and FGA/SGA dose were analyzed separately. *p* ≤ 0.05 was considered statistically significant in the logistic test.

In addition, the correlation between the PANSS scale and the PSP scale was analyzed to test for collinearity bias and to decide whether they should be analyzed separately. An aOR >1 was considered a higher scale rating among DIP patients after the adjustment of the confounding factor while aOR <1 was considered a lower scale rating with the presence of DIP after adjusting the confounding factor (Devanand et al., 1992). SPSS 23.0 (IBM, Akmon, New York, United States) was used for data analysis.

## 3 Results

### 3.1 Sample characteristics

We recruited 969 schizophrenia patients into this study, and the sociodemographic characteristics, clinical variables, current treatments, and scale ratings of all subjects are presented in Table 1. More men were included in this study (52.8%). The patients had a median age of 42.0 years, a duration of illness of 14.6 years, and 13.4 years of antipsychotic use. Most of the patients were taking SGA monotherapy (61.5%), and the rest were undergoing SGA polytherapy (38.5%). Among those patients taking polytherapy treatment, 30.3% took SGA + SGA polytherapy, while 8.2% took SGA + FGA polytherapy. The exact frequency of the different antipsychotics being used is shown in Supplementary Material S2. Among our subjects, 12.2% were on mood stabilizers and 6.4% on antidepressants. In total, 15.7% took anticholinergics, and among these, 34.2% of the subjects fitted the criteria of DIP. The median overall antipsychotic dosage was 5.8 CPZ 100 mg and the average high D2 receptor antagonistic effect antipsychotic dosage was 1.0 CPZ 100 mg. The average valproate dose was 51.1 ± 189.2 mg/d, and the median lithium dose was 5.2 mg/d. The median of the high and low/medium antipsychotic dosage **×** the years since the first prescribe antipsychotic, SGA/FGA dosage **×** the years since the first prescribe antipsychotic, and the clinical scale rating are shown in Table 1.

**TABLE 1:** Characteristics of the study subjects.

Sociodemographic characteristic	Value
Male sex, number (%)	512 (52.8%)
Age, mean (median)	42.0 (39.0)
Clinical variables	
Illness duration (median, IQR)	14.6 (25.3)
Disease characteristics, first episode (n, %)	210 (21.7%)
Duration of the current illness episode (median, IQR)	5.1 (5.5)
Years since the first prescribed antipsychotic (median, IQR)	13.4 (25.6)
PANSS positive (median, IQR)	7.0 (8.0)
PANSS negative (median, IQR)	16.0 (9.0)
PANSS disorganized (median, IQR)	6.0 (5.0)
PANSS excited (median, IQR)	5.0 (4.0)
PANSS depressed (median, IQR)	4.0 (3.0)
PANSS total score (median, IQR)	68.0 (35.0)
CDSS total score (median, IQR)	1.0 (3.0)
CGI-S (median, IQR)	4.0 (2.0)
PSP, socially useful activities (median, IQR)	3.0 (2.0)
PSP, personal and social relationships (median, IQR)	3.0 (2.0)
PSP, self-care (median, IQR)	1.0 (1.0)
PSP, disturbing and aggressive behavior (median, IQR)	1.0 (1.0)
PSP, total score (median, IQR)	60.0 (28.0)
Current treatment	
SGA (n, %) (Truong and Frei, 2019)	596 (61.5%)
SGA + SGA (n, %)	294 (30.3%)
SGA + FGA (n, %)	79 (8.2%)
High D2 receptor antagonistic antipsychotic (n, %)	375 (38.7%)
Mood stabilizer (n, %)	85 (12.2%)
Antidepressants (n, %)	62 (6.4%)
Anticholinergics (n, %)	152 (15.7%)
Valproate dose (mg, mean ± SD)	51.1 ± 189.2
Lithium dose (mg, median, IQR)	5.2 (63.4)
SGA dose (CPZ 100 mg, median, IQR)	5.2 (4.2)
FGA dose (CPZ 100 mg, median, IQR)	0.6 (0.1)
Overall antipsychotic dose (CPZ 100 mg, median, IQR	5.8 (4.2)
High D2 receptor antagonistic antipsychotic dose (CPZ 100 mg, median, IQR)	1.0 (3.0)
Low or medium D2 receptor antagonistic antipsychotic dose (CPZ 100 mg, median, IQR)	4.8 (6.5)
High D2 receptor antagonistic antipsychotic dose **×** years since the first prescribed antipsychotic (CPZ 100 mg **×** year, median, IQR)	2.7 (4.3)
Low or medium D_2_ receptor antagonistic antipsychotic dose **×** years since the first prescribed antipsychotic (CPZ 100 mg **×** year, median, IQR)	41.3 (145.3)
SGA **×** years since the first prescribed antipsychotic (CPZ 100 mg **×** year, median, IQR)	51.5 (142.5)
FGA **×** years since the first prescribed antipsychotic (CPZ 100 mg **×** year, median, IQR)	0.2 (0.2)

Abbreviation: PANSS, Positive and Negative Symptoms Scale; CDSS, Calgary Depression Rating Scale; CGI-S, clinical global impression-severity; PSP, Personal and Social Performance Scale; IQR, interquartile range

### 3.2 Comparison of clinical variables between DIP and non-DIP groups

Considering that all sociodemographic data and clinical data except valproate dose had a non-normal distribution verified by the K-S test (data not shown), the Mann–Whitney *U* test was used to compare all the data except valproate dose. The prevalence of DIP in this study was 19.1%. Compared with the non-DIP group, the DIP group was significantly older (Z = −5.655, *p* < 0.001), accompanied by longer illness duration (Z = −4.503, *p* < 0.001) and antipsychotic intake duration (Z = −4.781, *p* < 0.001). The DIP group contained more relapsing schizophrenia patients (X^2^ = 8.97, *p* = 0.003), and illness duration with the current episode was significantly longer (Z = −2.808, *p* = 0.005). DIP patients were prescribed more mood stabilizers with a threshold significance (X^2^ = 6.42, *p* = 0.01), and the valproate dose was significantly higher (T = −2.863, *p* = 0.005) (Madhusoodanan et al., 2010). There were more prescriptions for FGA (X2 = 3.12, *p* = 0.1), with a statistical threshold difference among the DIP group (0.05 < *p ≤* 0.1). The doses of FGA (Z = −1.840, *p* = 0.064) and high D2 receptor antagonistic antipsychotics (Z = −1.613, *p* = 0.093) were higher in the DIP group, with a threshold statistical difference. However, there was no significant difference in low/medium D2 receptor antagonistic antipsychotic dose and overall antipsychotic dose between the two groups. The value of high D2 receptor antagonistic antipsychotic dose **×** years since the first prescribe antipsychotic (Z = −4.548, *p* < 0.001), low/medium D2 receptor antagonistic antipsychotic dose **×** years since the first prescribe antipsychotic (Z = −3.301, *p* = 0.001), SGA **×** years since the first prescribe antipsychotic (Z = −4.504, *p* < 0.001), and FGA **×** years since the first prescribe antipsychotic (Z = −4.770, *p* < 0.001) were significantly higher in the DIP group. The comparison of sociodemographic data and clinical variables between the two groups is presented in Table 2.

**TABLE 2:** Comparison of clinical variables between DIP and non-DIP groups.

Covariate	Non-DIP group (*n* = 784)	DIP group (*n* = 185)	Z/T/X2	*p*
Sex (male, n, %)	410 (52.3%)	102 (55.1%)	0.48	0.513
Age (median, IQR)	40.0 (23.0)	53.0 (25.5)	−5.655	<0.001
Illness duration (median, IQR)	11.0 (24.7)	18.8 (26.0)	−4.503	<0.001
Years since the first prescribed antipsychotic (median, IQR)	9.5 (24.3)	5.7 (28.8)	−4.781	<0.001
Disease episodes (first episode, n, %)	169 (21.5%)	25 (13.5%)	8.97	0.003
Duration of the current episode (median, IQR)	0.9 (5.1)	5.1 (6.4)	−2.808	0.005
SGA (n, %)	489 (62.4%)	107 (57.8%)	1.30	0.28
SGA + SGA (n, %)	237 (30.2%)	57 (30.8%)	0.02	0.88
SGA + FGA (n, %)	58 (7.4%)	21 (11.4%)	3.12	0.10
High D_2_ receptor antagonistic antipsychotics (n, %)	296 (37.8%)	79 (42.7%)	1.54	0.24
Mood stabilizers (n, %)	60 (7.7%)	25 (13.5%)	6.42	0.01
Antidepressants (n, %)	48 (6.1%)	14 (7.6%)	0.52	0.50
Anticholinergics (n, %)	116 (14.8)	36 (19.5%)	2.46	0.12
Valproate dosage (mg, mean ± SD)	39.5 ± 160.9	100.0 ± 276.4	−2.863	0.005
Lithium dosage (mg, median, IQR)	5.0 (60.1)	5.3 (65.5)	−0.427	0.67
SGA dose (CPZ 100 mg, median, IQR)	5.0 (4.2)	6.0 (4.0)	−1.166	0.244
FGA dose (CPZ 100 mg, median, IQR)	0.2 (0.3)	0.7 (0.3)	−1.840	0.064
SGA × years since the first prescribed antipsychotic (CPZ 100 mg × year, median, IQR)	43.7 (135.4)	88.8 (158.3)	−4.504	<0.001
FGA × years since the first prescribed antipsychotic (CPZ 100 mg × year, median, IQR)	1.3 (0.2)	2.2 (0.4)	−4.770	<0.001
Overall antipsychotic dose (CPZ 100 mg, median, IQR)	5.2 (4.2)	6.7 (6.0)	−1.468	0.142
High D_2_ receptor antagonistic antipsychotic dose (CPZ 100 mg, median, IQR)	0.9 (2.4)	1.5 (4.0)	−1.613	0.093
Low or medium D_2_ receptor antagonistic antipsychotic dose (CPZ 100 mg, median, IQR)	4.3 (6.7)	5.2 (5.5)	−0.340	0.734
High D2 receptor antagonistic antipsychotic dose × years since the first prescribed antipsychotic (CPZ 100 mg × year, median, IQR)	1.4 (4.6)	4.2 (4.0)	−4.548	<0.001
Low or medium D2 receptor antagonistic antipsychotic dose × years since the first prescribed antipsychotic (CPZ 100 mg × year, median, IQR)	22.4 (118.2)	54.3 (169.7)	−3.301	0.001

Abbreviations: IQR, interquartile range; FGA, first-generation antipsychotics; SGA, second-generation antipsychotics. Actors in bold are included in the analysis of risk factors of DIP. The FGA/SGA model and high and low/medium D_2_ receptor medium antipsychotic models were analyzed separately.

The PANSS negative symptom scores (Z = −9.090, *p* < 0.001), the PANSS disorganized symptom scores (Z = −5.467, *p* < 0.001), and the PANSS total scores (Z = −5.556, *p* < 0.001) of DIP patients were significantly higher than the non-DIP group. CGI-S scores were also significantly higher in the DIP group (Z = −2.449, *p* = 0.014). The DIP group had higher PSP-useful activity scores (Z = −5.511, *p* < 0.001), PSP-personal and social relationships scores (Z = −4.146, *p* < 0.001), and PSP self-care subscale scores (Z = −6.096, *p* < 0.001), indicating the poorer social function of schizophrenia patients with DIP. The non-DIP group had significantly higher PSP-disturbing and aggressive behavior scores than the DIP group (Z = −1.961, *p* = 0.05). The non-DIP group had significantly higher PSP total scores (Z = −4.727, *p <* 0.001), indicating the poorer social function of schizophrenia patients with DIP. The comparison of clinical correlates and social function between the two groups is given in Table 3.

**TABLE 3:** Comparison of clinical correlates and social function between DIP and non-DIP groups.

	Non-DIP	DIP group (*n* = 185)	Z/X2	*p*
	Group (*n* = 784)			
PANSS positive (median, IQR)	7.0 (8.0)	7.0 (7.0)	−1.159	0.247
PANSS negative (median, IQR)	16.0 (9.0)	21.0 (21.0)	−9.090	<0.001
PANSS disorganized (median, IQR)	6.0 (5.0)	8.0 (8.0)	−5.467	<0.001
PANSS excited (median, IQR)	5.0 (4.0)	5.0 (5.0)	−1.430	0.143
PANSS depressed (median, IQR)	4.0 (3.0)	4.0 (4.0)	−0.909	0.153
PANSS total score (median, IQR)	66.0 (31.0)	77.0 (37.0)	−5.556	<0.001
CDSS total score (median, IQR)	1.0 (3.0)	1.0 (1.0)	−0.998	0.319
CGI-S (median, IQR)	4.0 (2.0)	4.0 (4.0)	−2.449	0.014
PSP, socially useful activities (median, IQR)	3.0 (2.0)	4.0 (4.0)	−5.511	<0.001
PSP, personal and social relationships (median, IQR)	3.0 (2.0)	3.0 (3.0)	−4.146	<0.001
PSP, self-care (median, IQR)	1.0 (1.0)	2.0 (2.0)	−6.096	<0.001
PSP, disturbing and aggressive behavior (median, IQR)	1.0 (1.0)	1.0 (1.0)	−1.961	0.05
PSP, total score (median, IQR)	60.0 (27.0)	51.0 (38.0)	−4.727	<0.001

Abbreviation: PANSS, Positive and Negative Symptoms Scale; CDSS, Calgary Depression Rating Scale; CGI-S, clinical global impression-severity; PSP, Personal and Social Performance Scale; IQR, interquartile range.

### 3.3 Risk factors and clinical correlates of DIP

A significant positive correlation was shown between FGA dosage and high D2 receptor antagonistic antipsychotic dosage (R = 0.141, *p* < 0.001, data not shown). Thus, the high and low/medium D2 receptor antagonistic antipsychotics model and FGA/SGA model were analyzed separately. After controlling for confounding factors, age (OR = 1.03, 95% CI = 1.02-1.04*,* and *p* < 0.001), high D2 receptor antagonistic antipsychotic dose (OR = 1.08, 95% CI = 1.01-1.16, and *p* = 0.032), and valproate dosage (OR = 1.01, 95% CI = 1.01-1.02*,* and *p =* 0.001) were defined as risk factors of DIP in the high and low/medium D2 receptor antipsychotic model. Subjects who had taken mood stabilizers had higher scores on item 1 (bradykinesia), item 3 (shoulder shaking), and item 9 (tremor) (Supplementary Material S3). In the FGA/SGA model, only age (OR = 1.03, 95% CI = 1.02-1.04, and *p* < 0.001) and valproate dose (OR = 1.01, 95% CI = 1.01-1.02, and *p* = 0.001) but not the dosage of FGA or SGA were significant risk factors of DIP. The risk factors of DIP derived from the two models are shown in Tables 4 and 5.

**TABLE 4:** Risk factors of DIP (high and low/medium D2 receptor antagonist antipsychotics).

Factor	B	SE	WALD	*p*-value	OR	95% CI
Age	0.030	0.005	30.319	<0.001	1.03	1.02-1.04
High D_2_ receptor antagonist antipsychotic dose	0.075	0.036	4.406	0.032	1.08	1.01-1.16
Valproate dose	0.001	<0.001	11.067	0.001	1.01	1.01-1.02
Constant	−2.967	0.280	112.148	<0.001	0.05	

**TABLE 5:** Risk factors of DIP (FGA/SGA antipsychotic model).

Factor	B	SE	WALD	*p*-value	OR	95% CI
Age	0.028	0.005	30.875	<0.001	1.03	1.02–1.04
Valproate dose	0.001	<0.001	10.884	0.001	1.01	1.01–1.02
Constant	−2.784	0.263	112.203	<0.001	0.06	

There was a significant co-linearity between the PSP subscale and the PANSS five-model subscale. Thus, a separate analysis of the PANSS model and PSP model was necessary (data not shown). Schizophrenia patients with DIP showed significant correlation with older age (OR = 1.02, 95% CI = 1.01–1.03, and *p* < 0.001), higher valproate dosage (OR = 1.01, 95% CI = 1.01–1.02, and *p* = 0.004), higher negative symptoms scores (OR = 1.09, 95% CI = 1.06–1.13, and *p* < 0.001), higher disorganized symptoms scores (OR = 1.08, 95% CI = 1.01–1.17, and *p* = 0.033), and lower excited symptom score (OR = 0.91, 95% CI = 0.86–0.97, and *p* = 0.002) in the PANSS scale model. In the PSP scale model, DIP patients showed significant correlation with older age (OR = 1.02, 95% CI = 1.01–.03, and *p* < 0.001), higher PSP-socially useful activity scores (OR = 1.27, 95% CI = 1.09–1.49, and *p* = 0.004), higher PSP self-care subscale scores (OR = 1.53, 95% CI = 1.28–1.82, and *p <* 0.001), lower PSP-disturbing and aggressive behavior scores (OR = 0.65, 95% CI = 0.52–0.81, and *p <* 0.001), and higher valproate dosage (OR = 1.01, 95% CI = 1.01–1.02, and *p =* 0.001). The clinical correlates of DIP derived from the two models are shown in Tables 6 and 7.

**TABLE 6:** Clinical correlates of DIP (PANSS scale).

Factor	B	SE	WALD	*p*-value	OR	95% CI
PANSS negative	0.089	0.015	35.637	<0.001	1.09	1.06–1.13
PANSS disorganized	0.076	0.035	4.540	0.033	1.08	1.01–1.17
PANSS excited	−0.090	0.030	9.410	0.002	0.91	0.86–0.97
Age	0.020	0.006	12.497	<0.001	1.02	1.01–1.03
Valproate dose	0.001	<0.001	8.247	0.004	1.01	1.01–1.02
Constant	−4.206	0.402	100.243	<0.001	0.02	

Abbreviation: PANSS, Positive and Negative Symptoms Scale.

**TABLE 7:** Clinical correlates of DIP (PSP scale).

Factors	B	SE	WALD	*p*-value	OR	95% CI
Age	0.023	0.006	17.388	<0.001	1.02	1.01–1.03
Valproate dose	0.001	<0.001	10.303	0.001	1.01	1.01–1.02
PSP, socially useful activities	0.236	0.082	8.381	0.004	1.27	1.09–1.49
PSP, self-care	0.425	0.090	22.468	<0.001	1.53	1.28–1.82
PSP, disturbing and aggressive behavior	−0.430	0.113	14.497	<0.001	0.65	0.52–.81
Constant	−4.206	0.402	100.243	<0.001	0.02	

Abbreviation: PSP, Personal and Social Performance Scale.

## 4 Discussion

In this study, we found a DIP prevalence of 19.1%. This was important because few real-world studies have reported the prevalence of DIP in the Chinese schizophrenia population. The prevalence of DIP was relatively low in our study considering most of the participants had taken SGA monotherapy, and the tolerated dose range of each antipsychotic was stipulated before the beginning of the study. Other published data showed that the prevalence of DIP ranged from 13.4% to 60% (Novick et al., 2010; Parksepp et al., 2016; Mentzel C. et al., 2017; Misdrahi et al., 2019). The wide range of DIP prevalence could be the result of many factors including differences in study populations, antipsychotic treatments, drug dosages, and diagnostic criteria*.*

To the best of our knowledge, this study is the first to show that it is not the FGA dosage but the high D2 receptor antagonist antipsychotic dose that correlates with the induction of DIP. The exact relationship between the antipsychotic dose and DIP remains unclear, however. Several previous clinical trials did not find a link between high daily doses of antipsychotics and DIP (Knol et al., 2012; Parksepp et al., 2016; Fountoulakis et al., 2017; Misdrahi et al., 2019), while other studies detected a significant correlation between oral dose of haloperidol (a typical antipsychotic with high antagonistic effect on D2 receptors), elevated plasma levels, and DIP (Devanand et al., 1992; Pelton et al., 2003). Meanwhile, the correlation between oral antipsychotic dose and TD was detected in some studies (Miller et al., 2005; Bakker et al., 2013). These phenomena can be explained by several factors. First, most of our population was treated with SGA monotherapy. The dosage of FGA in this study was relatively low, so statistically relevant conclusions about the effects of FGA on the induction of DIP cannot be drawn. Also, acute EPS, including DIP, may be more easily induced by antipsychotics with relatively high D2 receptor affinity compared with antipsychotic that have low/medium D2 receptor affinity antagonistic effect.

EPS and DIP are neurological side effects caused by blockade of the D2 receptor in the nigrostriatal dopamine system (Miyamoto et al., 2012). The induction of EPS positively correlated with D2 dopamine receptor occupancy because 75%–80% occupancy mediated significantly more EPS (Farde et al., 1992; Kasper et al., 1999). This study has also found that blockade of the D2 receptor on cholinergic interneurons and the consequent increase of acetylcholine signaling on the substantia nigra/striatum pathway significantly correlated with symptoms of haloperidol-induced parkinsonism, including motor-reducing and cataleptic effects (Kharkwal et al., 2016). Notably, DIP was only induced in mice injected with high D2 receptor antagonistic antipsychotic haloperidol but not with the atypical antipsychotic, clozapine. Correlation between the usage of a strongly binding D2 receptor antipsychotic and induction of EPS was also seen in the Chinese population sampled by our team for this study (Weng et al., 2019). A lower DIP prevalence for patients who took weaker D2 receptor antagonists compared to those who took a high D2 receptor antagonistic was also reported in a different study (Druschky et al., 2020). None of the prior drug exposure factors, including high D2 receptor antagonistic antipsychotic dose × years since the first prescribed antipsychotic, were not significant risk factors for DIP. However, we did not record the exact duration of use of each antipsychotic. The correlation between long-term drug exposure and DIP needs further investigation.

The induction of DIP was significantly correlated with age in our study, and several other studies have confirmed the relationship between old age and antipsychotic-induced movement disorder, but the outcome still remains controversial. Some researchers concluded that older age might predispose to DIP (Jiménez Jiménez et al., 1996; Savic et al., 2017; Druschky et al., 2020), while others found no significant correlation (Mentzel T. et al., 2017; Misdrahi et al., 2019). Our study did prove a possible correlation between age and DIP, but it could be explained by the fact that elderly people are more likely to suffer from movement disorders, including sclerosis, amyotrophy, orthopedic disorders, and other physical impairments that could bias the evaluation of SAS parameters, such as bradykinesia and arm dropping*.* Second, older individuals tend to have a lower ability to metabolize drugs, which could lead to higher plasma drug concentrations and a greater likelihood of adverse effects (Caligiuri et al., 1999; Uchida et al., 2009). We suggest that the prescription of high D2 receptor antagonistic antipsychotics at high dosage in elderly patients should be carefully monitored so that DIP onset can be detected and its progression stopped.

Valproate dose is also a risk factor for DIP, significantly correlated with psychiatric symptoms and poor social adaption. Among schizophrenia patients in the population, 90.5% received valproate monotherapy, 8.8% took mood stabilizers, 7.1% received lithium monotherapy, and 2.4% took both drugs. The induction of DIP and drug-induced tremor may occur with the administration of antiepileptic drugs, including valproate, but this phenomenon has frequently been ignored in the psychiatric field (Lautin et al., 1979; Wang et al., 2016). The average dose of valproate was 657.24 ± 258.29 mg/d, which is lower than the dosage previously reported to induce DIP. These phenomena may be explained by the use of antipsychotics monotherapy (Masmoudi et al., 2006; Silver and Factor, 2013). Valproate is an anticonvulsant drug that is commonly prescribed for the treatment of bipolar disorder, impulsive or violent behavior associated with psychiatric disease, or for concomitant treatment of drug-resistant schizophrenia (Huband et al., 2010; Wang et al., 2016). Valproate can enhance GABA activity and inhibit dopamine transmission in the substantia nigra, which contributes to the induction of EPS and cognitive impairment (Paladini et al., 1999; Rosenberg, 2007). Furthermore, Lei et al. (2017) showed that lithium could cause parkinsonism symptoms and cognitive disability by lowering tau protein and elevating nigral-cortical iron, which results in neurotoxicity through activation of the calcineurin/NFAT/Fas pathway (Lei et al., 2017). Valproate can also cause disorders of iron metabolism, thus inducing excessive oxidative stress, which is an important factor in the pathogenesis of Parkinson’s disease (Ounjaijean et al., 2011; Puspita et al., 2017). The mechanism of DIP induction by mood stabilizers is complicated and needs to be further explored. The rational application of valproate is critical and its discontinuation should be considered when it is likely to have an impact on cognition and social function in schizophrenia populations.

After controlling for confounding factors, DIP was found to be significantly correlated with more severe negative psychiatric symptoms, cognitive decline, and milder excitatory symptoms. Greater dysfunction in socially beneficial activities, self-care capabilities, and milder disturbing and aggressive behavior were also significantly correlated with DIP. In addition, age was also correlated with social dysfunctions. The results showed that the more severe negative symptoms and emotional apathy of DIP schizophrenia patients, after adjusting for confounding variables, could be among the root causes of DIP influencing both psychiatric symptoms and social functions of schizophrenia patients. Some clinical studies have proven this phenomenon (Heinz et al., 1998; Weng et al., 2019). Several hypotheses could explain these phenomenon of bad treatment outcome: 1) the consequence of drug-induced negative symptoms and drug-induced social dysfunction including antipsychotics or mood stabilizers (Artaloytia et al., 2006; Kim et al., 2019), 2) the dysfunction of the meso-cortical-limbic dopaminergic system caused by dopamine receptor blockade (Marchese and Pani, 2002; Molina et al., 2019), 3) dopamine neurotoxic effect of antipsychotics (Crowley et al., 2013; Rollema et al., 1994), 4) influence of age (Linke et al., 2015; Pujol et al., 2014). Overall, the mechanism behind this phenomenon is very complex. Considering that an effective treatment for these negative symptoms is lacking, the early identification of DIP and closer monitoring of antipsychotic drug administration to reduce the damage and dysfunction of the dopamine neuron pathway in the early stage is critically important.

### 4.1 Limitations

This study suffers from the limitation that we analyzed cross-sectional data without subsequently visiting the patients. The results of our study should be validated in a more suitable cohort. Our research cohort was a population of chronically hospitalized Chinese patients with relatively long illness duration. The number of outpatients and those with short illness duration was relatively small. The DIP patients in our population may include some with tardive parkinsonism, which has been detected in several studies (Savitt and Jankovic, 2018) and may have different risk factors with more complex mechanisms. Future studies should establish distinct criteria to distinguish tardive DIP from acute DIP.

### 4.2 Conclusions and future perspectives

From this study, we learned that age, treatment with high D2 receptor antagonistic antipsychotics, and the valproate dose are the main risk factors for DIP. DIP was significantly correlated with psychiatric symptoms and social dysfunction in Chinese schizophrenia patients. Old age was also a key factor related to psychotic symptoms and ability to function socially. The rational application or discontinuation of valproate is necessary because of its direct impact on psychiatric symptoms and social functioning of schizophrenia patients. Early intervention and treatment of DIP could improve the prognosis and social capabilities of schizophrenia patients. Future studies are needed to verify our findings in cohort studies and determine how broadly our results can be applicable to others. Lastly, the bias of tardive parkinsonism should be excluded.

## Data Availability

The datasets presented in this article are not readily available because of further data analysis. Requests to access the datasets should be directed to HL lhlh_5@163.com.
