# Supplementary material for: Risk factors, clinical correlates, and social functions of Chinese schizophrenia patients with drug-induced parkinsonism: A cross-sectional analysis of a multicenter, observational, real-world, prospective cohort study
Source: Front Pharmacol. 2023 Mar 3;14:1077607. doi: 10.3389/fphar.2023.1077607 (PMC10020528; doi:10.3389/fphar.2023.1077607)
Supplement: Supplementary file 1 [file Table1.DOCX]

| High D2 receptor antagonistic effect antipsychotic | Low/medium D2 receptor antagonistic effect antipsychotic |
| --- | --- |
| Risperidone, Ziprasidone, Paliperidone, Sulpiride, Chlorpromazine, Haloperidol, Fluphenazine, Pentafluridol | Quetiapine, Amisulpride, Clozapine, Olanzapine, Aripiprazole |

**Supplementary 1. The exact category of high and low/medium D2 receptor antagonistic effect antipsychotics**
