# Supplementary material for: Risk factors, clinical correlates, and social functions of Chinese schizophrenia patients with drug-induced parkinsonism: A cross-sectional analysis of a multicenter, observational, real-world, prospective cohort study
Source: Front Pharmacol. 2023 Mar 3;14:1077607. doi: 10.3389/fphar.2023.1077607 (PMC10020528; doi:10.3389/fphar.2023.1077607)
Supplement: Supplementary file 2 [file Table2.DOCX]

**Supplementary 2. Frequency of different antipsychotics being used**

| **SGA** | Non-DIP Group (n = 784) | DIP Group  (n = 185) |
| --- | --- | --- |
| Risperidone | 179 | 58 |
| Quetiapine | 54 | 13 |
| Amisulpride | 70 | 10 |
| Clozapine | 146 | 38 |
| Olanzapine | 235 | 661 |
| Paliperidone | 61 | 7 |
| Aripiprazole | 231 | 58 |
| Ziprasidone | 13 | 8 |
| **FGA** |  |  |
| Sulpiride | 9 | 55 |
| Chlorpromazine | 27 | 13 |
| Haloperidol | 6 | 2 |
| Fluphenazine | 14 | 4 |
| Pentafluridol | 0 | 2 |

**Abbreviation:** FGA: first-generation antipsychotics, SGA: second-generation antipsychotics.
