# Supplementary material for: Risk factors, clinical correlates, and social functions of Chinese schizophrenia patients with drug-induced parkinsonism: A cross-sectional analysis of a multicenter, observational, real-world, prospective cohort study
Source: Front Pharmacol. 2023 Mar 3;14:1077607. doi: 10.3389/fphar.2023.1077607 (PMC10020528; doi:10.3389/fphar.2023.1077607)
Supplement: Supplementary file 3 [file DataSheet1.docx]

**Supplementary 3.**


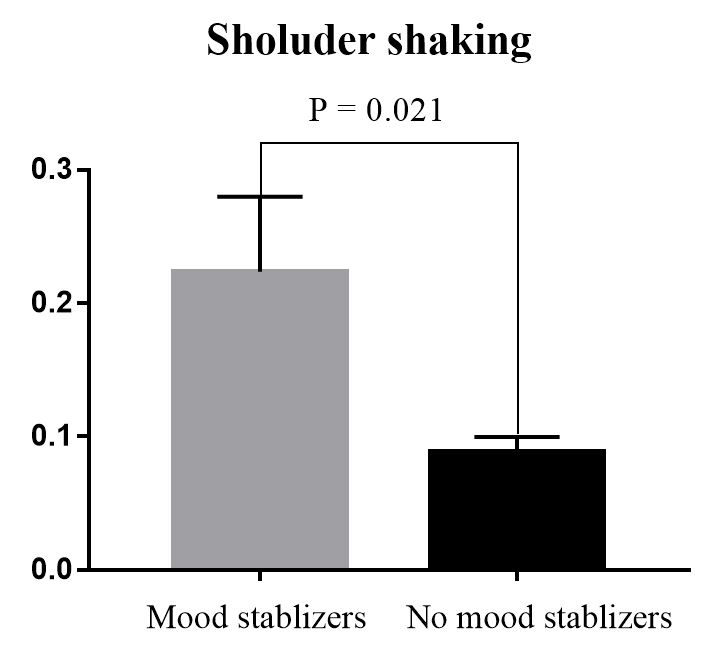

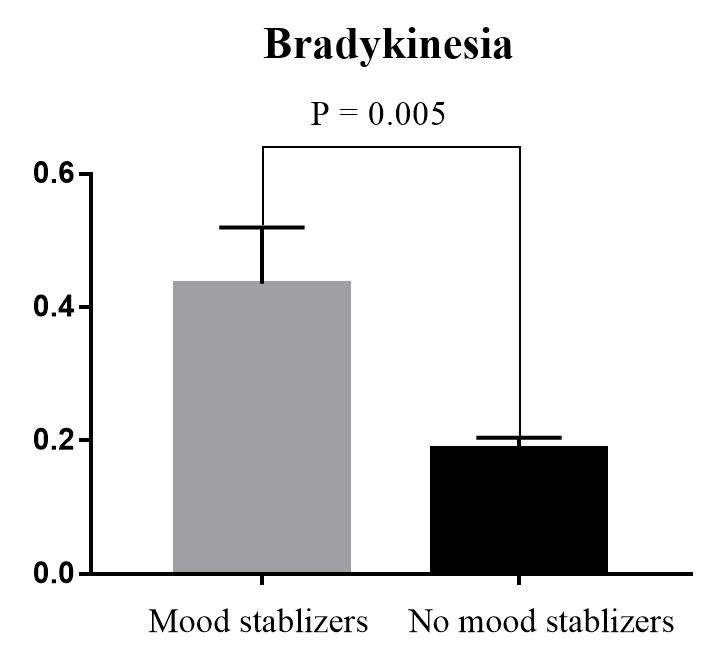
Figure A Figure B


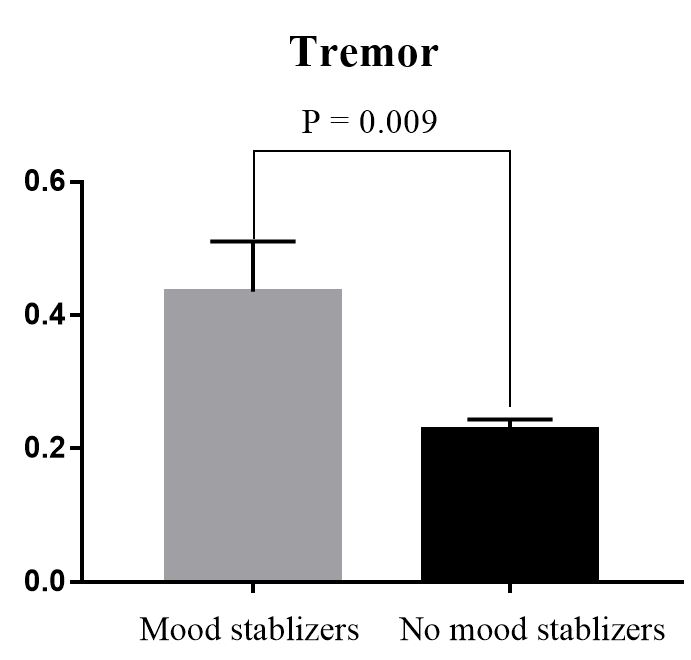
Figure C

**Figure A:** The comparison of the bradykinesia score between those taking mood stabilizers and those not taking mood stabilizers.

**Figure B:** The comparison of shoulder shaking between those taking mood stabilizers and those not taking mood stabilizers.

**Figure C:** The comparison of tremor score between those taking mood stabilizers and those not taking mood stabilizers.
